# A case of infected schwannoma mimicking malignant tumor

**DOI:** 10.1186/s12957-016-1058-3

**Published:** 2016-12-07

**Authors:** Mamer S. Rosario, Norio Yamamoto, Katsuhiro Hayashi, Akihiko Takeuchi, Shinji Miwa, Hiroyuki Inatani, Takashi Higuchi, Hiroyuki Tsuchiya

**Affiliations:** 1 Department of Orthopaedic Surgery, Kanazawa University School of Medicine, 13-1 Takara-machi, Kanazawa, Ishikawa 920-8640 Japan; 2 Department of Orthopaedics, East Avenue Medical Center, East Avenue, Diliman, Quezon City, 1101 Metro Manila Philippines

**Keywords:** Schwannoma, Infection, Group B *Streptococcus*, Abscess, Tumor

## Abstract

**Background:**

Infected schwannoma has been reported, this being one of the four cases published in the literature. Infected schwannoma has proven to be a tough diagnostic challenge to the treating tumor surgeon, mimicking infectious entities and most essentially, a malignant tumor.

**Case presentation:**

The authors report the case of a 64-year-old male with a soft tissue mass in his right gluteal area that presented initially with right leg pain, then later with signs of inflammation on the tumor area. Magnetic resonance imaging (MRI), computed tomography (CT), and thallium-201 scintigraphy studies confirm the presence of soft tissue mass which had continuity with sciatic nerve, with subsequent serial MRI findings suggesting tumor enlargement with cystic degeneration. Increased level of C-reactive protein (CRP) was observed before surgery. During an open biopsy upon tissue sampling, exudates with necrotic tissue were seen. Increased level of CRP and necrotic change suggested the possibility of malignant tumor. Histopathological diagnosis was schwannoma, and group B *Streptococcus* was detected by culture. After the confirmation of infected schwannoma, enucleation of the tumor was performed.

**Conclusions:**

The report concludes that establishment of a benign pathology is essential when presented with similar clinical findings prior to definitive enucleation of an infected schwannoma.

## Background

Schwannoma is an unusual benign tumor arising from the nerve sheath comprising of Schwann cells [1]. Common locations include the head and neck, the flexor surfaces of the upper and lower extremities, the posterior mediastinum in the thorax, and the trunk [2]. The benign tumor is commonly 2 to 4 cm in size but can become large in deep tissues like the posterior mediastinum or retroperitoneum [2]. Larger tumors can present with pain and neurological symptoms clinically, and cystic formation can be seen due to degenerative changes histopathologically [3].

Schwannomas mostly arise adjacent to a peripheral, or less commonly an intracranial nerve [1]. These tumors occur principally in adults and equally in males and females [1] but are less common in children [4]. The lesions are round and quite smooth and regular in appearance and are tightly attached to the neural elements [1].

These benign tumors are occasionally glandular in structure [5]. But histologically, schwannoma tissue has some remarkable features that are not only quite distinctive but denote major diagnostic elements. These features include Antoni A patterns in which the cells are elongated as are their nuclei; [6] Antoni B patterns in which the cells are irregular and the tissue between them are markedly hyalinized; [3, 6, 7] and Verocay bodies which show large collections of fibrous elements between the Antoni A cells [8, 9]. In addition, the tumor tissue may contain Wagner–Meissner bodies with xanthomatous changes [10]. All the cells stain weightily with S100 [6, 9, 10].

Infected schwannoma does happen but is extremely rare. After a thorough review and to the best of the authors’ knowledge, this is one of the only four cases of infected schwannoma reported in the literature. The other three were cases of infected gastric [11], retroperitoneal [12], and glossal [13] schwannomas. But more interesting than the rarity of infected schwannoma is the diagnostic challenge that a similar vagueness of its clinical presentation poses to the treating tumor surgeon. Schwannoma has been reported in the literature mimicking infectious disease entities such as psoas abscess [14, 15], nerve abscess of leprosy [16–18], Bartholin’s gland abscess [19], and acute appendicitis [20]. It has also been reported as presenting concomitantly with, and hidden by, an infectious pathology [21, 22]. A similar clinical presentation can also be thought of a malignant tumor undergoing rapid growth and subsequent necrotic change and cystic degeneration within the mass [23–25]. Lastly, cases of malignant transformation of a schwannoma have been documented in the literature [26–28], some being radiation-induced [29, 30] and sometimes even to an angiosarcoma [31–35].

The authors report the case of a 64-year-old male who received steroid pulse therapy at another hospital and presented with an infected schwannoma in his right gluteal area. This report underscores the importance of establishing a benign tumor diagnosis by final histopathology prior to definitive enucleation of the mass when presented with findings suggestive of an infected schwannoma.

## Case presentation

A 64-year-old male had suffered from right leg pain accompanying an enlarging mass in his right gluteal area. The patient received steroid pulse therapy because of sudden deafness at another hospital at 5 months prior to consult. Right leg pain appeared 1 month later. There was no neurological deficit.

**Fig. 1:**
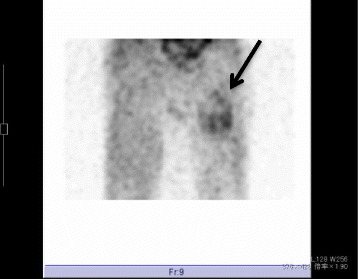
Thallium-201 scintigraphy showing uptake at the area of tumor (see *arrow*)

**Fig. 2:**
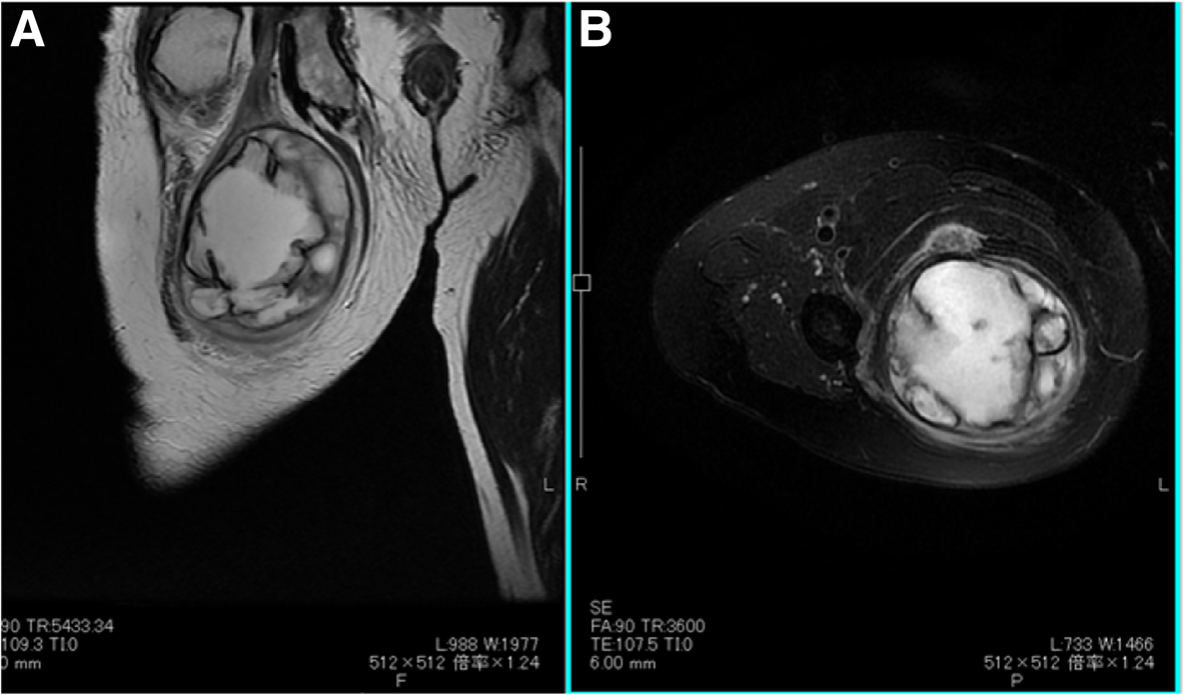
**a**, **b** T2-weighted (**a**) and T2-weighted fat-suppressed (**b**) MR images of mass showing the enlargement and homogenous appearance of contents with high signal intensities, indicating cystic degeneration

A firm, soft tissue mass with poor mobility was palpated deep to the area of the right gluteal crease. Before surgery, Tinel’s sign was positive. Computed tomography (CT) showed no phleboliths or foci of calcification within the tumor, and thallium-201 scintigraphy showed accumulation on the area of the mass (see Fig. 1). Magnetic resonance imaging (MRI) showed enlargement and homogenous contents with high T2-weighted signal intensities, suggesting cystic degeneration (see Fig. 2a, b). Elevated C-reactive protein (CRP) and increased white blood cells (WBC) were observed before surgery. Differential diagnoses comprised of schwannoma and sarcomas including malignant peripheral nerve sheath tumor with necrotic change.

**Fig. 3:**
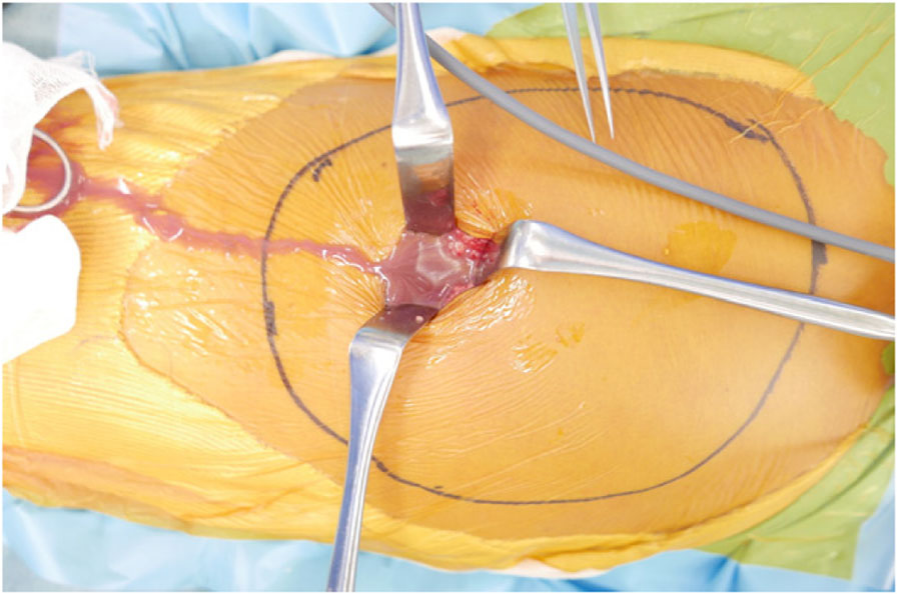
Pus coming out of tumor during open biopsy procedure

Before excision of the tumor, an open biopsy was performed. During the open biopsy, exudate with necrotic tissue was seen (see Fig. 3). A part of the tumor tissue and the exudate was obtained. Pathologic findings show spindle-shaped, S100-positive cells arranged in fascicles with no nuclear atypia, with scattered lymphocytes and degenerative necrotic changes. Culture of the exudate yielded group B *Streptococcus*, and postoperative antibiotics were shifted to intravenous cefazolin, at a dose 500 mg every 8 hours. Taken together, the mass was diagnosed as infected schwannoma.

**Fig. 4:**
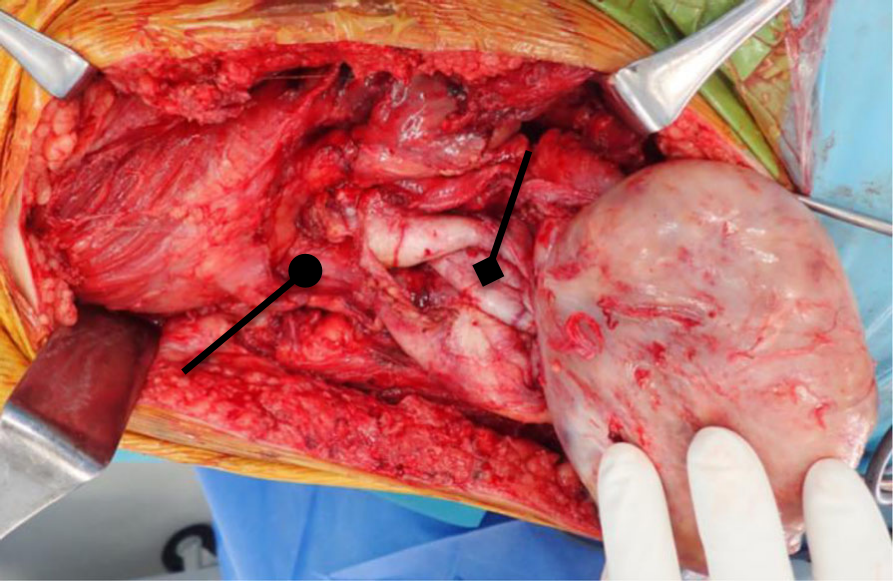
Capsule (*diamond arrow*) and mass seen attached to the sciatic nerve (*round arrow*)

After the confirmation of the existence of bacteria and pathological diagnosis released 5 days following the open biopsy, enucleation was imperative because CRP and WBC counts were persistently elevated (see Table 1). Enucleation of the mass with debridement and irrigation was performed on the day of release of the final histopathological report. The mass had a thickened capsule and was seen attached to the sciatic nerve (see Fig. 4). Nerve stimulator was used during surgery to prevent postoperative motor deficits. Following tumor removal, debridement of surrounding devitalized tissues and copious irrigation with saline solution was done.

**Table 1:** CRP and WBC determinations (with neutrophil counts) before and after open biopsy

	8 days before	2 days before	1 day after	3 days after	5 days after (DOE)	7 days after	10 days after
WBC (×10^3^)	12.68 (H)	11.45 (H)	10.15 (H)	8.55	7.54	7	7.15
PMNs (%)	80.4 (H)	79 (H)	**	79.9 (H)	73.8 (H)	67 (H)	62
CRP (mg/dl)	18.7 (H)	10.8 (H)	**	11.5 (H)	8 (H)	6.9 (H)	5.5

*PMNs* polymorphonuclear leukocytes, *CRP* C-reactive protein, *H* high, *WBC* white blood cells, *DOE* date of enucleation

** PMNs and CRP had no results on this day

**Fig. 5:**
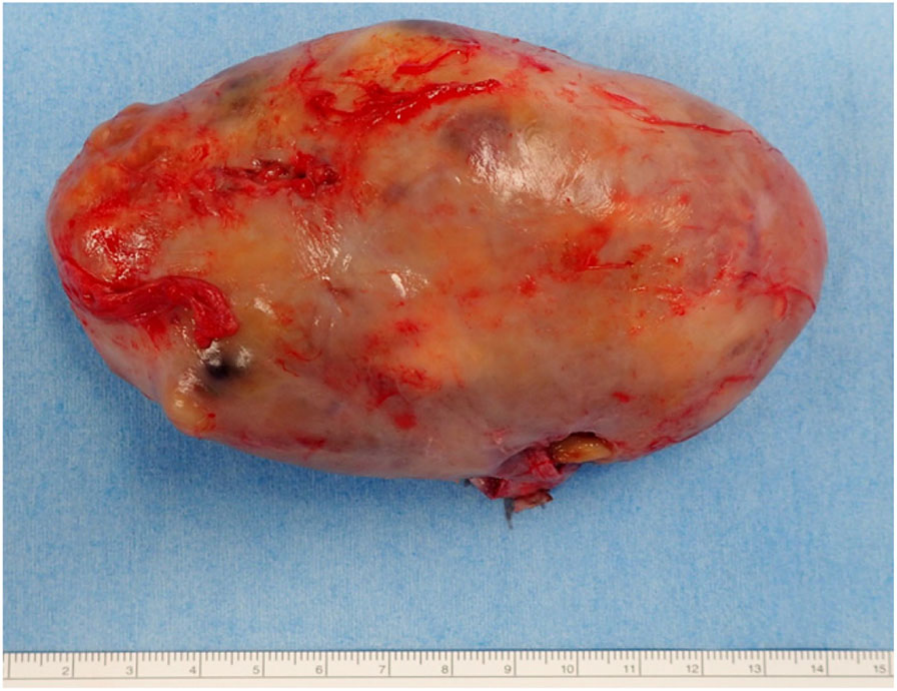
Schwannoma extirpated by enucleation measuring 11 cm × 6.5 cm × 5 cm

**Fig. 6:**
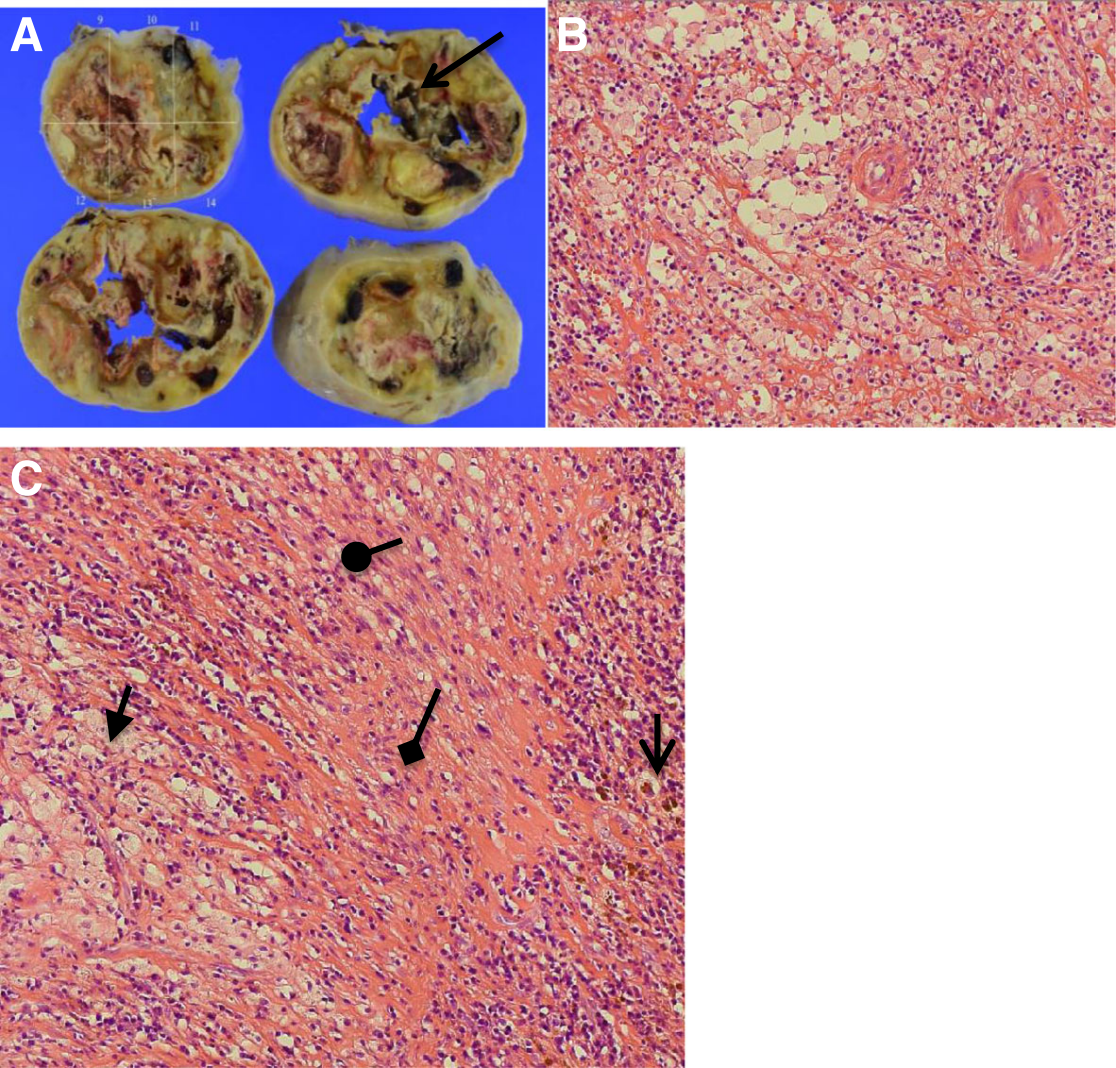
**a**–**c**. **a** Gross specimens show degeneration within tumor (*arrow*). **b** Hematoxylin and eosin staining under high magnification shows cystic necrotic changes with abundance of lymphocytes and macrophages. **c** Antoni A (*triangle arrow*) and Antoni B cells (*diamond arrow*) with hemosiderin deposits (*open arrow*) and tumor cells arranged in bundled sheets (*round arrow*) seen under high magnification

The mass was round, firm, and brownish-yellow in color, measuring 11 cm × 6.5 cm × 5 cm (see Fig. 5). Gross (see Fig. 6a) and histopathologic findings with hematoxylin and eosin staining (see Fig. 6b, c) revealed cystic necrotic tumor degeneration with abundance of macrophages and lymphocytes accompanying Antoni A and B cells, consistent with a diagnosis of schwannoma with degenerative changes. Culture yielded group B *Streptococcus*. The patient was continually administered intravenous cefazolin for 1 week, and oral cefdinir for 1 month. Successive CRP determinations decreased (see Table 1), and the pain had been improved after the surgery.

## Conclusions

Schwannomas larger than 5 cm in diameter have a tendency to undergo cystic degenerative change [36, 37]. Moreover, the cyst formation itself makes the tumor even larger [38]. The proposed mechanism for degeneration is the vascular insufficiency resulting from the increase in size—the poor vascular supply results to necrosis, hemorrhage, blood resorption, and hyaline degeneration within the tumor that subsequently leads to cyst formation [39, 40]. This cystic degenerative change is also commonly seen in ancient schwannomas, first described by Ackerman and Taylor in 1951, that is comprised entirely of Antoni type B tissue [41]. It is considered that degenerative change producing poor vascularization within the tumor provides the nidus for hematogenous infection of a schwannoma.

Malignant tumors, however, due to their inherent rapid growth, can undergo the same internal necrosis [23–25] and, therefore, present comparably as large schwannomas undergoing cystic degenerative change. A differential diagnosis also worth mentioning is the inflammatory subtype of undifferentiated pleomorphic sarcoma, which mimics an abscess clinically and presents with constitutional symptoms including fever and malaise in addition to an elevated CRP and erythrocyte sedimentation rate (ESR) [23]. Malignant transformation of a schwannoma has been documented in many published reports [26–28, 31–35]. Seeing exudates with necrotic tissue during the open biopsy made it difficult to determine the diagnosis not only because of the rarity of an infected schwannoma [11–13] but more so, of the possibility of a malignant pathology. The authors thought it prudent to wait for the final histopathological report before deciding on a definite surgical plan to avoid “undertreating” the case in the event that the diagnosis turned out to be a malignancy.

Previous reports cited at the beginning of this paper show how tricky a schwannoma diagnosis can be when presented clinically with a backdrop similar to that of an infectious pathology [14–22]. Although a homogenous signal on T2-weighted MRI accompanying clinical signs of infection in this case favors an abscess over a malignant pathology, the reports imply that schwannoma must be a competing differential diagnosis in such case. An open biopsy, therefore, prior to a conclusive surgical procedure, should be done. The authors have decided to perform an open biopsy because in the present case, there was cystic change of the mass, and the cystic change made it difficult to obtain tumoral tissue by needle biopsy even if the biopsy is performed with the guidance of ultrasonography or CT. The infected gastric schwannoma case of Euanorasetr and Suwanthanma [11] could have avoided a Billroth 2 procedure if only an open biopsy was initially done that could have ruled out their preoperative working diagnosis of gastric lymphoma. Two other gastric schwannoma cases [42, 43], also misdiagnosed as malignancies preoperatively, ended up with partial gastrectomies instead of just local tumor extirpations [42]. Although these were cases of “overtreatment” and not “undertreatment”, anatomical and corresponding functional losses from the gastric surgeries are undesirable for a patient. Similarly, missing out on a malignancy and subsequently “undertreating” a tumor is never a desirable occurrence for the treating tumor surgeon. Therefore, the authors of this case report deemed the open biopsy prior to definitive surgery very crucial.

Singh et al. [12], in their case of a large, infected retroperitoneal schwannoma, did not perform debridement and irrigation following removal of the tumor, although the postoperative recovery was purportedly uneventful. In the present case, the authors performed debridement and irrigation of devitalized peritumoral tissue following tumor enucleation. With the use of nerve stimulator, removal of nonviable tissue with the preservation of neural structures was safely accomplished. The authors considered doing a debridement and irrigation important in the present case to prevent recurrence of the infection.

The predominance of lymphocytes in both the open biopsy and final histopathological reports in the present case, in a supposedly acute bacterial infection, elucidates the role of lymphocytes during ongoing, nonresolving infection. Attention is traditionally placed on polymorphonuclear leukocytes (PMNs) in acute bacterial infection, with the influx of PMNs unquestionably observed at the initial phase and their accumulation peaking by the 2nd day following onset of acute bacterial infection [44]. In the present case, both the biopsy and final histopathological analyses were performed at least a week since CRP determinations were already elevated. Lymphocytes disappear once PMNs begin to accumulate, in response to PGD_2_ working through its DP1 receptor. However, beyond the period of intense inflammation when PMNs start to disappear, lymphocytes are observed repopulating the site of inflammation [44]. Rajakariar et al. [44] have found lymphocytic repopulation occurring not only during progression of the acute infection but also during and beyond resolution, and have concluded that lymphocytic repopulation during acute inflammation does not primarily signal resolution, but serves as the body’s preparation to mediate responses to a superinfection where lymphocytes are observed to predominate. The timing of the histopathological studies shall explain the lymphocytic repopulation and the absence of PMNs or bacterial bodies noted in both the biopsy and final histopathological reports for the present case, for which an imminent superinfection must have been anticipated.

Group B *Streptococcus* (GBS) disease has emerged as a major cause of invasive infections in adults for the past three decades [45–49]. In a recent population-based surveillance among California adults from 1995 to 2012 [50], the incidence has increased from 5.8 to 8.3 cases/100,000 persons (*p* < 0.001), mostly among men aged more than 40 years (*p* < 0.002). One study identified increasing age as being associated with risk even after adjusting for specific chronic illnesses which are more frequent among older persons [47], and the clinical presentation of invasive GBS disease among adults most often takes the form of bacteremia with no identified source [49, 51].

Taken altogether, the patient in the present case, with a history of steroid pulse therapy and possibly of steroid-induced immunosuppression, could have contracted the infection from a silent GBS bacteremia. When presented with an infected tumor in an adult allegedly immunocompromised, it is prudent that GBS disease must be ruled out. Cefdinir, an oral cephalosporin relatively new in the market, has been shown to have broad-spectrum activity including against GBS [52]. The authors, therefore, tapered the intravenous antibiotic cefazolin to oral cefdinir, considering the latter to be an ideal oral antibiotic for a patient allegedly immunocompromised. The authors have also extended the regimen from the usual 2 weeks to 1 month, as prophylaxis for immune augmentation given the background of possible steroid-induced immunosuppression [53].

The authors conclude that establishment of a benign tumor diagnosis by final histopathological analysis is essential when presented with similar clinical findings prior to definitive tumor enucleation of an infected schwannoma.

## Abbreviations

CRP: C-reactive protein
CT: Computed tomography
ESR: Erythrocyte sedimentation rate
GBS: Group B *Streptococcus*
H: High
MRI: Magnetic resonance imaging
PMN: Polymorphonuclear leukocytes
WBC: White blood cells
